# Glucose-6-Phosphate Dehydrogenase of Trypanosomatids: Characterization, Target Validation, and Drug Discovery

**DOI:** 10.4061/2011/135701

**Published:** 2011-04-04

**Authors:** Shreedhara Gupta, Mariana Igoillo-Esteve, Paul A. M. Michels, Artur T. Cordeiro

**Affiliations:** ^1^Research Unit for Tropical Diseases, de Duve Institute, TROP 74.39, Avenue Hippocrate 74, 1200 Brussels, Belgium; ^2^Department of Chemistry, Heritage Institute of Technology, Chowbaga Road, Anandapur, Kolkata 700107, India; ^3^Laboratory of Experimental Medicine, Université Libre de Bruxelles, Route de Lennik 808, CP 618, 1070 Brussels, Belgium; ^4^Laboratory of Biochemistry, Université Catholique de Louvain, Brussels, Belgium; ^5^Laboratório Nacional de Biociências (LNBio), Centro Nacional de Pesquisas em Energia e Materiais (CNPEM), Caixa Postal 6192, 13083-970 Campinas, SP, Brazil

## Abstract

In trypanosomatids, glucose-6-phosphate dehydrogenase (G6PDH), the first enzyme of the pentosephosphate pathway, is essential for the defense of the parasite against oxidative stress. *Trypanosoma brucei, Trypanosoma cruzi*, and *Leishmania mexicana* G6PDHs have been characterized. The parasites' G6PDHs contain a unique 37 amino acid long N-terminal extension that in *T. cruzi* seems to regulate the enzyme activity in a redox-state-dependent manner. *T. brucei* and *T. cruzi* G6PDHs, but not their *Leishmania* spp. counterpart, are inhibited, in an uncompetitive way, by steroids such as dehydroepiandrosterone and derivatives. The *Trypanosoma* enzymes are more susceptible to inhibition by these compounds than the human G6PDH. The steroids also effectively kill cultured trypanosomes but not *Leishmania* and are presently considered as promising leads for the development of new parasite-selective chemotherapeutic agents.

## 1. Introduction

The family Trypanosomatidae, belonging to the order Kinetoplastida, contains a large number of species, distributed over several genera. All known members of the Trypanosomatidae family are parasites, infectious to humans and other mammals, other vertebrates, insects and plants. The human-infective trypanosomatids are grouped in species of two genera, *Trypanosoma* and *Leishmania*. They are responsible for a wide spectrum of diseases in tropical and subtropical countries. Two subspecies of *Trypanosoma brucei*, *T. b. rhodesiense*, and *T. b. gambiense*, are responsible for different forms of Human African Trypanosomiasis or sleeping sickness, an endemic disease in over 250 distinct foci in rural areas of 36 sub-Saharan African countries. The currently estimated number of cases is 50,000–70,000 with 17,000 new infections annually and 60 million people at risk [1, 2]. The parasite is transmitted between human and/or other mammalian hosts by the tsetse fly. The complex life cycle of this extracellular parasite includes a procyclic form present in the midgut of the insect vector and a bloodstream form present in the blood of the mammalian hosts [3].

*Trypanosoma cruzi* is responsible for Chagas' disease in most countries of Latin America. It is estimated that 11–18 million people are infected; 13,000 deaths are reported annually and about 100 million people are at risk [4]. The parasites are transmitted by blood-sucking reduviid bugs. After infection, the metacyclic trypomastigotes invade host cells where they proliferate as the so-called amastigote forms and, after differentiation into bloodstream trypomastigotes, they infect more cells, notably of heart muscle, and alimentary track tissue [1, 5, 6].

Different species of *Leishmania* cause a variety of clinical symptoms, collectively called Leishmaniases. These diseases may involve cutaneous and mucocutaneous lesions, often causing severe debilitating wounds, or life-threatening visceral diseases in which vital organs are affected. The diseases threaten about 350 million people in 88 countries in tropical and subtropical parts of the world. An estimated 12 million people are currently infected with about 1-2 million new cases occurring annually. *Leishmania* transmission occurs via the byte of sandflies which inject metacyclic promastigote parasites into the skin. These forms enter macrophages where they reside as multiplying amastigotes within the phagolysosomes [7].

Sleeping sickness, Chagas' disease and visceral leishmaniasis can have a fatality rate as high as 100% if left untreated or not treated properly [1, 8]. But treatment with drugs currently available is highly unsatisfactory [9, 10]. Most drugs have low efficacy and adverse side effects. Moreover the emergence of drug resistance is a continuous concern. Therefore, and because of the lack of efficacious vaccines, the discovery and development of effective drugs, nontoxic, affordable and easy to administer to the affected populations in the resource-poor areas is an urgent need.

Despite the tremendous progress made in recent decades in understanding the biochemistry and molecular biology of trypanosomatid parasites [11–14], chemotherapeutic treatment of the diseases has seen limited progress. 

A currently common strategy for drug discovery against any parasitic diseases is to identify essential metabolic pathways associated with the parasites. In trypanosomatids, several enzymes involved in various metabolic processes have been characterized and established as promising drug targets [14, 15]. Among these validated targets is glucose-6-phosphate dehydrogenase (G6PDH; EC 1.1.1.49), a key enzyme of the pentosephosphate pathway.

## 2. The Role of the Pentosephosphate Pathway and Glucose-6-Phosphate Dehydrogenase

**Figure 1:**
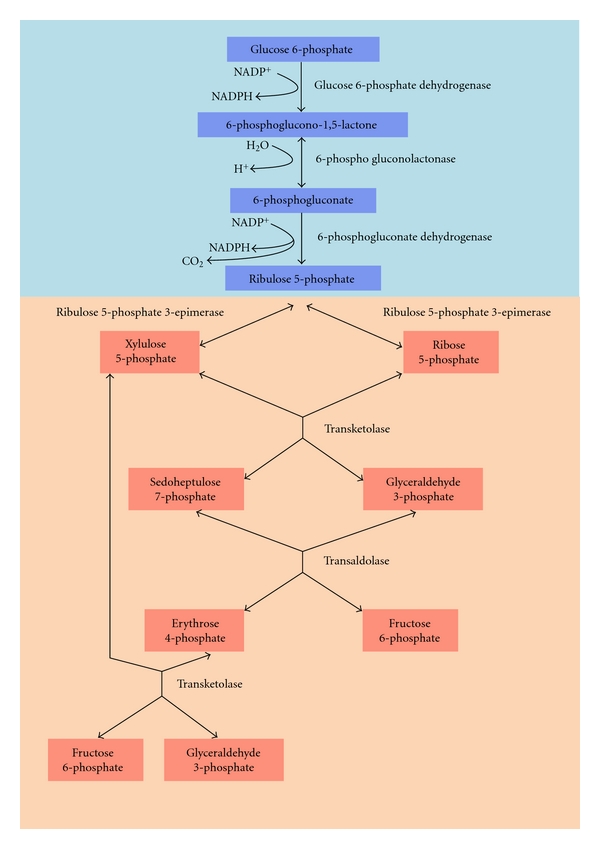
Schematic representation of the pentosephosphate pathway.

In most organisms glucose is metabolized through two major pathways: the glycolytic and the pentosephosphate pathway (PPP) [16]. Whereas glycolysis serves for ATP production and to produce metabolites for use in a large variety of anabolic or further catabolic processes, the PPP can be divided in two successive phases with different functions. The first phase, called oxidative branch, generates reducing power under the form of NADPH and the second one, also known as nonoxidative or sugar interconversion branch, involves a series of reversible nonoxidative reactions leading to the conversion of the 5-carbon sugar resulting from the first phase into other metabolites (Figure 1). The oxidative branch comprises three enzymes: G6PDH, 6-phosphogluconolactonase, and 6-phosphogluconate dehydrogenase (6PGDH), whose successive activities convert glucose 6-phosphate (G6P) into ribulose 5-phosphate, with the concomitant production of NADPH by both dehydrogenases and CO_2_ release by the decarboxylation of the 6-phosphogluconate by 6PGDH [17]. The NADPH is used by the cell as a reducing agent in a variety of biosynthetic processes as well as for the defense against reactive oxygen species by keeping glutathione in its reduced state [18]. Glutathione is the major endogenous cellular antioxidant, participating directly in the neutralization of free radicals and reactive oxygen compounds, as well as maintaining exogenous antioxidants such as vitamins C and E in their reduced, active forms.

The nonoxidative branch comprises ribose-5-phosphate isomerase, ribulose-5-phosphate epimerase, transketolase and transaldolase, which, by isomerase and epimerase reactions and the transfer of two- and three-carbon units, form a variety of intermediates including ribose 5-phosphate, required for nucleotide synthesis, and erythrose 4-phosphate a precursor of aromatic amino acids and vitamins [19].

Depending on the cell's needs, the PPP can function partially or as a whole cycle by which one of the possible products, fructose 6-phosphate, is converted back into G6P, to enter the oxidative branch again. In addition, the PPP and glycolytic pathway are connected by sharing some intermediates such as G6P, fructose 6-phosphate and glyceraldehyde 3-phosphate.

G6PDH is the first committed enzyme of the PPP. Although most organisms have other means to produce cellular NADPH, notably by the enzymes isocitrate dehydrogenase and malic enzyme, the generally important role of G6PDH in it is illustrated by the severity of G6PDH deficiency in humans. The deficiency may lead to nonimmune hemolytic anemia triggered by oxidative stress as a result of infections or exposure to chemicals such as present in medication or certain foods [20]. G6PDH deficiency is the most common human enzyme defect, occurring in more than 400 million people worldwide [21].

## 3. Glucose-6-Phosphate Dehydrogenase in Trypanosomatids

The presence of G6PDH activity in trypanosomatids was first demonstrated in 1959 by Raw in *T. cruzi* epimastigotes [22] and in 1962 by Ryley in both cultured procyclic and bloodstream form *T. b. rhodesiense *[23]. A few years later, studies with labeled glucose by Mancilla and colleagues [24–26] suggested that the PPP is functional in some *T. cruzi* strains and *Leishmania *species and, much later, Voorheis and coworkers [27] confirmed the presence of a classical PPP in *T. brucei* by determining the specific activities of all its enzymes in both procyclic and bloodstream forms. Interestingly, no activities of ribulose-5-phosphate epimerase and transketolase were detectable in the bloodstream form and only low activity was found for transaldolase, suggesting an important differential repression of the nonoxidative branch in this life-cycle stage [27]. For some of the PPP enzymes, like G6PDH and 6-phosphogluconolactonase, a dual subcellular distribution was later found in both bloodstream and procyclic form trypanosomes. While their main enzymatic activity was present in the cytosol, approximately 40 and 10%, respectively, of their activity was associated with glycosomes [28, 29]. Glycosomes are the peroxisome-like organelles in Kinetoplastida which uniquely harbour the majority of the enzymes of the glycolytic pathway [30]. These organelles, which number between 5 and 10 in *Leishmania* amastigotes and approximately 65 in bloodstream form *T. brucei*, may contain enzymes from a variety of different pathways [31–33], with the glycolytic enzymes being most notable and comprising even up to 90% of the organelles' protein content as observed in bloodstream form trypanosomes [33–35].

A functional PPP has also been unambiguously demonstrated in *T. cruzi* epimastigotes by measuring the CO_2_ production from radiolabeled glucose [36]. Moreover, the importance of the PPP was demonstrated by a challenge with methylene blue, mimicking oxidative stress, which caused a twofold increase of the flux through the PPP. All enzymes of the pathway were identified by activity measurements in the four major developmental stages of this parasite. The activities were shown to be predominantly cytosolic, with a possible small glycosomal component for most of them.

In a comparable study, the presence of a functional PPP was also demonstrated in promastigotes of *Leishmania mexicana*, by its twofold stimulation by methylene blue. Each of the classical PPP enzymes was identified and its specific activity measured [37]. Furthermore, it was shown that glucose serves as a source for nucleic acid synthesis, an indication that, in these parasites, the PPP plays also a role in the conversion of glucose into ribose 5-phosphate for nucleotide biosynthesis. Also in *Leishmania*, the PPP has probably a dual subcellular localization, although it has only been demonstrated so far for the transketolase that is predominantly present in the cytosol of promastigotes, but also with a significant component associated with the glycosomes [38]. The presence of the PPP in glycosomes of the three trypanosomatid species is further supported by the presence of peroxisomal-targeting signals (PTSs) in a number of enzymes of both the oxidative and nonoxidative branches [31, 39] and—for *T. brucei*—by the results of proteomic analyses [31, 32].

Whereas in almost all organisms, glutathione, reduced by NADPH produced predominantly in the PPP, is the major intermediate in the defense against reactive oxygen species, in trypanosomatids a different thiol redox component is being used, trypanothione, or N^1^,N^8^-bis-gluthionyl-spermidine. Several enzymes involved in trypanothione metabolism have been detected both in the cytosol and glycosomes [31].

Preliminary enzymatic studies on G6PDH have been performed with the partially purified enzyme from *T. cruzi* [40, 41] and *T. brucei* [28], but detailed characterization of G6PDH of each of the three trypanosomatid species was only performed after the genes had been identified and used to produce the recombinant proteins. Genomic analysis revealed one gene copy per haploid genome in *T. brucei* [29] and *L. mexicana *[42] and several genes located on three different chromosomes of *T. cruzi* clone CL Brener [43, 44]. Two of them were classified as pseudogenes, while the others were clustered in three groups of nearly identical (98%) coding regions but with considerably different noncoding flanking sequences. The amino acid sequences of the functional G6PDHs of three trypanosomatids are 64 to 69% identical and share about 50% identity with the human enzyme.

**Figure 2:**
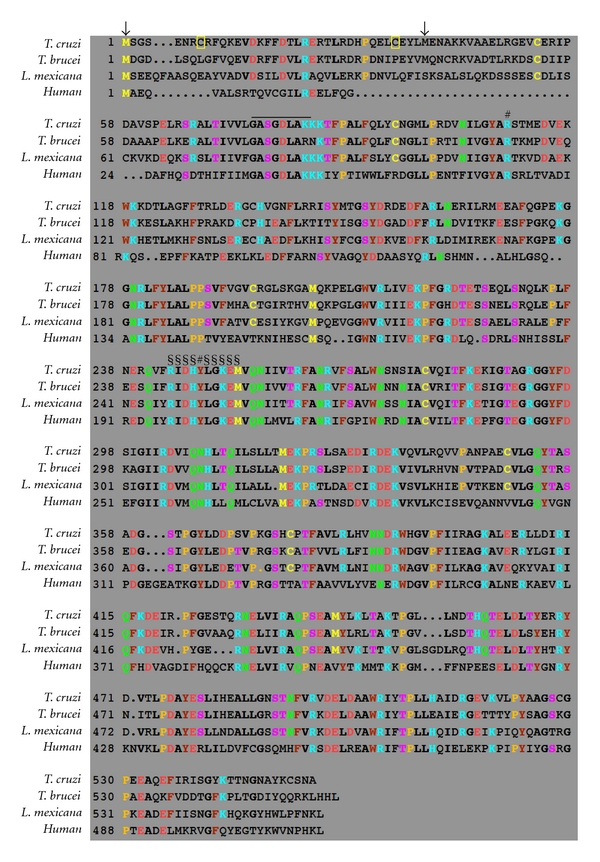
G6PDH amino acid sequence alignment. The predicted amino acid sequences of *T. cruzi *G6PDH-*long *(DQ408239, sharing the highest identity with its* T. brucei* counterpart), *T. brucei* G6PDH-*long* (CAC07816), *L. mexicana *G6PDH (AAO37825), and Human G6PDH (AAL27011) were aligned using the *CLUSTALW* software. The first and second candidate initiator methionines in the *T. cruzi *and *T. brucei *sequences are indicated with arrows. The two regulatory cysteines of the *T. cruzi *G6PDH are boxed in yellow. Overlined, cofactor binding site; ^§^G6PDH signature (residues belonging to the substrate binding site); ^#^residues involved in substrate and cofactor binding.

The *T. brucei* and *T. cruzi *G6PDH gene sequences both have two possible start codons, 111 bp apart (Figure 2), while in *Leishmania* species only a unique start codon was found corresponding to the first one of the *Trypanosoma* genes. The region between the two start codons codes for a usual 37 amino acid N-terminal extension only present in the trypanosomatid G6PDHs. The long form of the *T. brucei* and *T. cruzi* enzymes code for polypeptides of 557 and 555 residues, respectively, while the *L. mexicana* G6PDH polypeptide encompasses 562 residues. Western blot analysis, performed with an antiserum raised against the N-terminal peptide of the *T. cruzi* G6PDH, showed that mainly the long form of the enzyme is expressed in all four life-cycle stages of this parasite [43]. Whether the long form of* T. brucei* G6PDH is also produced remains to be determined. Duffieux and coworkers [29] proposed that an ORF starting at the first ATG codon would lead to an mRNA with an unusually short 5′ untranslated region.

The *L. mexicana* G6PDH, the short form of the *T. brucei* enzyme and both the long and short form of *T. cruzi* G6PDH have been expressed with an N-terminal His-tag in *Escherichia coli*, purified and kinetically characterized [29, 43, 45, 46]. The kinetic mechanism has been studied, under conditions which were similar for all enzymes, and appeared to follow for each of them a bi-bi-ordered pattern [45, 46]. The values of the kinetic constants are presented in Table 1.

Interestingly, it was shown that the long and short form of *T. cruzi *G6PDH present several differences [43]. Experiments performed independently from those for which the results are presented in Table 1, revealed differences in the apparent *K*_*m*_ values for G6P. While the purified recombinant long and short *T. cruzi* G6PDH had apparent *K*_*m*_ values of 189.9 and 98.4 *μ*M, respectively, that of the partially purified enzyme from parasites was 288 *μ*M. In contrast, the apparent *K*_*m*_ values for NADP^+^ did not differ. Additionally, both the natural enzyme and the long recombinant G6PDH, but not the short form, were inactivated by reducing agents such as dithiothreitol, *β*-mercaptoethanol, and reduced glutathione. This effect, reminiscent to the redox-state-dependent activity regulation observed for G6PDH and some other enzymes from chloroplasts and cyanobacteria [47], was attributed to the presence of two Cys residues in the N-terminal peptide [43]. The corresponding N-terminal region of G6PDH of *Leishmania* spp. and the possible extension of the *T. brucei* enzyme do not contain these Cys residues (Figure 2). These observations suggest that among the trypanosomatid G6PDHs studied, only the activity of the *T. cruzi* enzyme is regulated by the redox state of the cell, in a similar way as reported for the enzyme from chloroplasts and cyanobacteria. Indeed, kinetic studies with the two purified recombinant forms showed that the long form was 4-fold more sensitive for inhibition by the product NAPDH than the short one, suggesting that its activity is regulated by the NADP^+^/NADPH ratio in the cell compartment where the enzyme is located, similarly as has been proposed for the enzyme from *Anacystis nidulans* [48].

**Figure 3:**
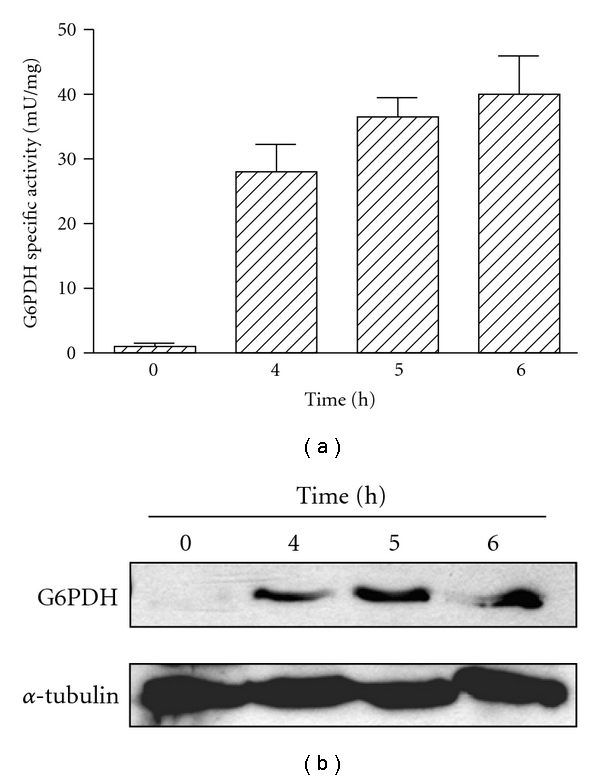
*T. cruzi *G6PDH expression and specific activity are enhanced by oxidative stress in metacyclic trypomastigotes. Parasites were incubated for 4 to 6 h in the presence of 70 *μ*M H_2_O_2_. Samples for enzymatic activity measurements and western blots were taken before (T0) and after the H_2_O_2_ addition. (a) G6PDH specific activity. The results are means ± SE of quadruplicates. (b) Autoradiography of a western blot corresponding to metacyclic trypomastigotes cell-free extracts (30 *μ*g of total protein/lane). *α*-Tubulin was used as loading control. (Figure created with data from [43].)

**Figure 4:**
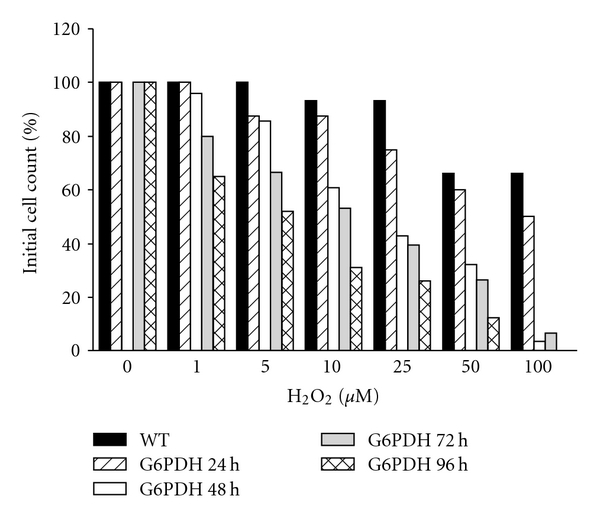
G6PDH is important for defense against oxidative stress in bloodstream form *T. brucei*. Wild-type (WT) trypanosomes and cells in which partial depletion of G6PDH was induced by RNAi were grown in regular HMI-9 medium. After growth for different periods of time as indicated, cells were collected by centrifugation and resuspended in non-reducing medium (i.e., without *β*-mercaptoethanol and cysteine) and the cell density was determined. Cell suspensions were then incubated for 1.5 h in the presence of different concentrations of H_2_O_2_ after which the cell density was determined again.

The importance of G6PDH in the defense against oxidative stress has been demonstrated for both the *T. cruzi* [43] and *T. brucei* enzyme (SG and PM, unpublished; see below). Incubation of *T. cruzi* with H_2_O_2_ modified the G6PDH expression and specific activity in the cell (Figure 3). In metacyclic trypomastigotes, the life-cycle stage naturally exposed to reactive oxygen species (ROS) produced by the mammalian host, the peroxide exposure enhanced the G6PDH expression as well as the specific activity of the enzyme. Conversely, peroxide treatment in epimastigotes, a life-cycle stage not naturally encountering such a host response, repressed the G6PDH expression and activity. In bloodstream forms of *T. brucei* G6PDH expression has been knocked down by tetracycline-inducible RNA interference (RNAi) [45]. Cells partially depleted from G6PDH showed enhanced sensitivity to different concentrations of H_2_O_2_ compared to wild-type trypanosomes (Figure 4).

## 4. Glucose-6-Phosphate Dehydrogenase Is a Genetically Validated Drug Target in Bloodstream Form **T. brucei**

As stated before, most organisms can produce NADPH not only by the action of the two dehydrogenases of the PPP—G6PDH and 6PGDH—but also via isocitrate dehydrogenase and malate dehydrogenase to cope with G6PDH deficiencies. However, trypanosomatids depend only upon malic enzyme [49] and G6PDH for their cytosolic NADPH production. In these parasites the isocitrate dehydrogenase is compartmentalized inside the mitochondrion and, in species of *Trypanosoma* but not *Leishmania*, it has also been predicted as a glycosomal enzyme [39].

**Figure 5:**
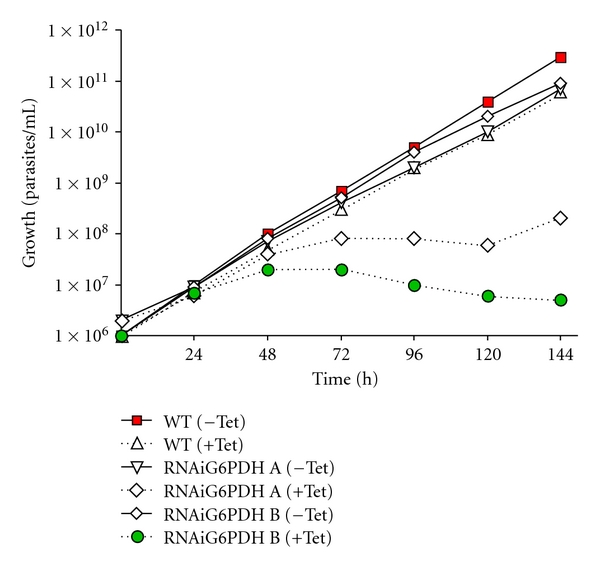
Genetic validation of G6PDH as a drug target in bloodstream form *T. brucei*. Cumulative growth of wild-type (WT) trypanosomes and two independent cell lines (RNAiG6PDH A and B). In the absence of RNAi induction (−Tet), WT and RNAi parasites grow at equal rates; in the presence of the RNAi inducer tetracycline (+Tet), WT cells grow at a normal rate, while trypanosomes of the RNAi-cell lines die after 48 h. (Figure created with data from [45].)

As mentioned above, *T. brucei* bloodstream form G6PDH RNAi cell lines have been created [45]. To determine if inhibition of G6PDH (and consequently the flux through the downstream located PPP enzyme 6PGDH) was sufficient to affect the trypanosome growth, a cell line in which RNAi induction led to a considerable reduction in G6PDH expression was studied. In these cells 24 h of RNAi induction led to a mild decrease in G6PDH, while 48 h of knockdown induced a substantial reduction in G6PDH protein, as determined by western blot analysis. The decrease in G6PDH expression was paralleled by an impairment in the trypanosomes' growth rate. This effect was observed between 24 and 48 h after the addition of tetracycline to the culture medium. Beyond 48 h of RNAi induction, G6PDH depleted cells started to die (Figure 5). Parasites containing the RNAi construct, but grown in the absence of tetracycline, showed a similar growth rate as wild-type cells grown in the presence or absence of tetracycline. The decrease of the growth rate correlated with the initiation of the RNAi induction and a simultaneous decrease in the protein level, suggesting that G6PDH plays an important role in growth and survival of the bloodstream form trypanosomes. The possibility that the observed phenotype was a consequence of an RNAi-off-target effect cannot be excluded, but it was considered to be unlikely since the G6PDH sequence is unique and no other highly similar gene was identified in the *T. brucei* genome database.

These RNAi experiments genetically validated G6PDH as a drug target in bloodstream forms of *T. brucei* and suggested that the NADPH produced by other enzymes than G6PDH is not sufficient to deal with oxidative stress experienced during normal or stress conditions (Figure 4).

## 5. Inhibition of Trypanosomatid G6PDH by Steroids

Inhibition of mammalian G6PDH by intermediates of the steroid-hormones biosynthesis was discovered 50 years ago by Mark and Banks [50]. They demonstrated a highly specific and uncompetitive inhibitory effect of dehydroepiandrosterone (DHEA) and derivatives on human G6PDH. These steroid molecules did not inhibit the homologous enzyme of either yeast or spinach. Moreover they did not affect the activity of mammalian 6PGDH and isocitrate dehydrogenase. During the following 35 years, it was a generally accepted notion that only G6PDH from mammalian cells was inhibited by steroid hormone derivatives [51]. As a consequence of such a generalization, the inhibition of G6PDH from lower eukaryotes, like trypanosomatids and other human parasites, was neglected. Observations that DHEA or derivatives of it were effective against infections of *Cryptosporidium parvum* [52], *Plasmodium *species [53], *Schistosoma mansoni* [54], and *T. cruzi *[55] were attributed to a stimulation of the host's immune response by the steroids. Nonetheless, in recent years it was shown that the steroids also affect growth of cultured parasites such as *Entamoeba histolytica* [56] and *Taenia crassiceps* [57], suggesting that they could have a direct effect on these organisms.

**Figure 6:**
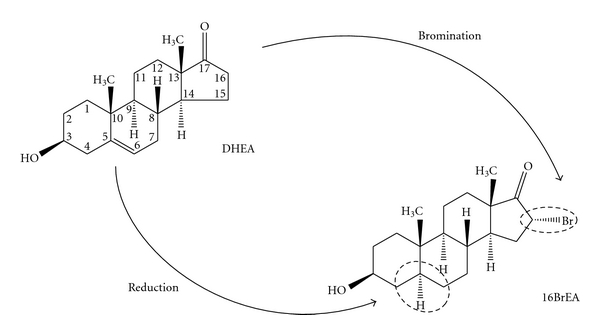
Steroid inhibitors of *Trypanosoma* G6PDH. Two modifications used to increase the inhibitory potency of steroids to G6PDH are indicated: reduction of the double bond between carbons 5 and 6 in dehydroepiandrosterone (DHEA) which leads to epiandrosterone (EA) and the bromination at position 16 which converts DHEA and EA into 16BrDHEA and 16BrEA, respectively.

Recently, Cordeiro et al. [45] confirmed these findings by showing that steroids kill *in vitro* grown *T. brucei* bloodstream forms with ED_50_ values in the micromolar range. They additionally demonstrated that *T. brucei *G6PDH, the first nonmammalian enzyme, was effectively inhibited by DHEA and epiandrosterone (EA). Similar observations were subsequently made for *T. cruzi* G6PDH [46]. Although, DHEA itself failed to decrease the growth rate of cultured *T. cruzi*, its brominated derivatives 16BrDHEA and 16BrEA (Figure 6), which are more potent inhibitors of G6PDHs, presented ED_50_ values in the micromolar range, comparable to values reported for benznidazole [58], the currently available drug in use for treatment of Chagas' disease. Curiously, DHEA and EA did not inhibit *L. mexicana *G6PDH and had no effect on the growth rate of cultured promastigote parasites. This observation was indirect evidence for the specific inhibition of G6PDH by DHEA in trypanosomes.

## 6. G6PDH Is the *In Situ* Target of Human Steroids with Trypanocidal Action

Recently, the mechanism by which DHEA and EA kill *T. brucei* bloodstream forms has been evaluated, by exploiting the fact that these compounds showed no inhibitory effect on the recombinant *L. mexicana *G6PDH. Inhibitors were tested on the cell growth of a trypanosome clone that has been created to also express a transgene encoding this *L. mexicana* enzyme [59]. Whereas wild-type bloodstream form *T. brucei* showed a dose-dependent killing by DHEA and EA with ED_50_ values of 41.8 ± 2.1 *μ*M and 21.4 ± 1.6 *μ*M, respectively, the *T*. *brucei *(*Lm*G6PDH) transgenic parasites showed no growth inhibition whatsoever by the two compounds, even at concentrations up to 100 *μ*M [59]. Thus, transfection of *T. brucei* bloodstream form parasites with *Lm*G6PDH could rescue the trypanosomes from being killed by DHEA and EA. This result confirms that the toxic effect of DHEA and EA on the parasites is uniquely due to the inhibition of their G6PDH.

## 7. Conclusions and Perspectives

Our research has validated the key PPP enzyme G6PDH as a target for new drugs to be developed against trypanosomes. Oxidative insults induce G6PDH expression and enhance its specific activity, while the partial depletion of this enzyme by RNAi makes the parasites vulnerable to oxidative stress. Prolonged depletion of G6PDH from cultured bloodstream form *T. brucei* resulted even in death of the parasites. Whether this was only due to an effect on the redox metabolism or also on the synthesis of metabolic intermediates to be used for biosynthetic processes still needs to be addressed.

Steroids derived from DHEA are potent uncompetitive inhibitors of *Trypanosoma* G6PDH, and indeed kill the parasites by *in situ* inactivating this enzyme. The uncompetitive nature of this inhibition is particularly relevant. Contrary to the much more often observed competitive inhibition, the increase of substrate concentration, resulting from this process, will not overcome the inhibition but may rather lead to an increase of metabolic intermediates to toxic levels [60]. The molecular mechanism by which the steroids inhibit the G6PDH of human, *Trypanosoma* and other lower eukaryotes including several human parasites, while they do not affect the activity of the enzyme from plants, yeasts, and *Leishmania* is not yet known. To analyze the differences between the *Trypanosoma* and human G6PDH's steroid binding site and the binding mechanism of these molecules, cocrystallization of the enzymes with steroids and the determination of their crystal structure is needed. To date, no three-dimensional structure of a trypanosomatid G6PDH is available, but crystallization studies are presently ongoing and, when successful, may open new possibilities for the design and synthesis of a different class of molecules with even higher inhibitory potency and selectivity for the parasite G6PDH.

Since these steroids inhibit *Trypanosoma* G6PDHs at much lower concentrations than the mammalian G6PDH, they are promising leads for the development of new drugs for treatment of African sleeping sickness and Chagas' disease. The next steps in the development of drug candidates against these diseases will involve the selection of compounds with potent and highly selective inhibitory activity on cultured parasites *versus* human cells, and the evaluation of their efficacy, bioavailability, and toxicity in infected animal models.

The lack of inhibition of the *Leishmania* G6PDH by DHEA and its derivatives is puzzling. It is feasible that this is due to a single but crucial substitution in the enzyme that prevents the binding of these compounds. Screening of a steroid library against the available recombinant *L. mexicana *G6PDH may help to identify potent uncompetitive inhibitors with *in vitro* anti-*Leishmania *spp. activity.

As mentioned above, steroids also impaired the growth of other parasites, that is,* Taenia crassiceps* and *Entamoeba histolytica*, as well as the parasitaemia of *Plasmodium falciparum* and *P. berghei*, *Cryptosporidium parvum*, and *Schistosoma mansoni*. It is still necessary to determine if G6PDH is also in these cases the main target of the steroid molecules. This will open new perspectives for discovery of drugs also against the diseases caused by these parasites.

Concerning the trypanosomatid-borne diseases, the availability of recombinant G6PDH for each of the three parasites makes also possible an alternative strategy, that is, using these enzymes for high-throughput screening of large libraries of drug-like compounds. This approach may lead to very wide range of inhibitors potentially exploitable for antiparasitic treatment.

## Figures and Tables

**Table 1:** *T. brucei* (Tb), *T. cruzi* (Tc), and *L. mexicana* (Lm) G6PDH kinetic constants for an ordered bi-bi-reaction mechanism.

Source	*K*_ G6P_	*K*_ NADP_^+^	V_1_	*k*_ cat_	*K*_ iG6P_
	(*μ*M)	(*μ*M)	(nmoles of NADPH·s^−1^)	(s^−1^)	(*μ*M)
Tb	57.8 ± 2.4	9.4 ± 0.4	36.2 ± 1.5	16.4 ± 0.6	47.6 ± 1.9
Tc	206.0 ± 4.2	22.5 ± 1.2	77.7 ± 2.5	57.1 ± 1.9	105.3 ± 4.6
Lm	74.5 ± 3.0	12.1 ± 0.5	31.2 ± 1.2	22.2 ± 0.9	86.4 ± 3.5

Data from Cordeiro et al. [45, 46].

## Abbreviations

G6P: Glucose 6-phosphate
G6PDH: Glucose-6-phosphate dehydrogenase
6PGDH: 6-phosphogluconate dehydrogenase
PPP: Pentosephosphate pathway
PTS: Peroxisome-targeting signal
RNAi: RNA interference
ROS: Reactive oxygen species.
